# *SELL* and *GUCY1A1* Gene Polymorphisms in Patients with Unstable Angina

**DOI:** 10.3390/biomedicines10102494

**Published:** 2022-10-06

**Authors:** Damian Malinowski, Magda Zawadzka, Krzysztof Safranow, Marek Droździk, Andrzej Pawlik

**Affiliations:** 1 Department of Pharmacokinetics and Therapeutic Drug Monitoring, Pomeranian Medical University, 70-111 Szczecin, Poland; 2 Department of Experimental and Clinical Pharmacology, Pomeranian Medical University, 70-111 Szczecin, Poland; 3 Department of Biochemistry and Medical Chemistry, Pomeranian Medical University, 70-111 Szczecin, Poland; 4 Department of Physiology, Pomeranian Medical University, 70-111 Szczecin, Poland

**Keywords:** SELL, GUCY1A1, polymorphism, unstable angina

## Abstract

Acute ischaemia is mostly caused by the rupture of an unstable atherosclerotic plaque in a coronary artery, resulting in platelet accumulation and thrombus formation, which closes the lumen of the coronary vessel. Many different factors can cause atherosclerotic plaques to occlude the lumen of a coronary artery, including factors that increase vascular inflammation and blood platelet aggregation, as well as genetic factors. L-selectin is an adhesion molecule encoded by the human *SELL* gene, playing an important role in leukocyte adhesion to the endothelium and the development of inflammation. Guanylate cyclase 1 soluble subunit alpha 1 (*GUCY1A1*) is a gene that affects vasoreactivity and platelet function, thereby influencing thrombotic processes and the risk of developing thrombotic lesions in the coronary vessels. In *SELL* and *GUCY1A1* genes, several polymorphisms have been detected, which may affect gene expression. The aim of our study was to assess the association between the *SELL* rs2205849 and rs2229569 and *GUCY1A1* rs7692387 polymorphisms with the risk of acute coronary syndromes in the form of unstable angina pectoris, and the association between these polymorphisms and selected clinical parameters affecting the risk of developing ischemic heart disease. The study included 232 patients with unstable angina. The diagnosis of unstable angina was achieved by a typical clinical presentation and confirmation of significant coronary artery lumen stenosis (>70%) during coronary angiography. There were no statistically significant differences in *GUCY1A1* rs7692387 and *SELL* rs2205849 and rs2229569 polymorphism distribution between the total study and the control groups. However, when only analysing patients over 55 years of age, we found a decreased frequency of the *GUCY1A1* rs7692387AA genotype (AA vs. GA + GG, OR: 0.07; 95% CI: 0.01–0.78) and an increased frequency of the *SELL* rs2205849 CC genotype (CC vs. TC + TT *p* = 0.022) and *SELL* rs2229569 AA genotype (AA vs. GA + GG *p* = 0.022) in patients with unstable angina. Our results suggest that the *SELL* rs2205849 and rs2229569 and *GUCY1A1* rs7692387 polymorphisms are not risk factors for unstable angina in the Polish population. The *GUCY1A1* rs7692387 polymorphism may increase the risk of unstable angina in patients younger than 55 years, while the *SELL* polymorphisms rs2205849 and rs2229569 may increase the risk of unstable angina in patients older than 55 years in the Polish population.

## 1. Introduction

Acute coronary syndromes (ACS) comprise a group of clinical entities with a similar pathophysiological mechanism leading to sudden myocardial ischaemia. These include unstable angina (UA) and acute myocardial infarction (MI). Acute ischaemia is mostly caused by the rupture of an unstable atherosclerotic plaque in a coronary artery, resulting in platelet accumulation and thrombus formation, which closes the lumen of the coronary vessel [1,2]. A number of different factors can cause the coronary artery lumen to be occluded by atherosclerotic plaques: local inflammation within the plaque, rupture or erosion of the plaque, the presence of an intracoronary thrombus and increased vasoreactivity [3]. Inflammation is involved in the pathogenesis of atherosclerosis, taking place within the vascular plaque, requiring the recruitment and adhesion of circulating inflammatory cells and their transendothelial migration [4]. Numerous adhesion molecules and other mediators that modify platelet function are involved in this process.

L-selectin is an adhesion molecule belonging to the selectin family. The expression of L-selectin has been found in leukocytes, mainly neutrophils, and it plays an important role in the initial phases of leukocyte adhesion to the endothelium [5]. Increased levels of L-selectin have been found in various diseases, such as ischaemic heart disease, vascular diseases and cancers [6,7,8]. L-selectin is encoded by the human *SELL* gene, located at 1q24-q25 [9]. Variants of this gene may be genetic risk factors for atherosclerosis-related and inflammatory diseases [10,11,12]. The −642C > T polymorphism (rs2205849) in the promoter region is one of the most frequently studied polymorphisms in the L-selectin gene. The T allele has been shown to be associated with the increased expression of L-selectin [13], while the 725C > T polymorphism (rs2229569) results in the substitution of proline to serine at codon 213, leading to altered leukocyte–endothelial interactions [14].

Guanylate cyclase 1 soluble subunit alpha 1 (*GUCY1A1*) is a gene that affects vasoreactivity and platelet function, thereby influencing thrombotic processes and the risk of developing thrombotic lesions in the coronary vessels. Previous studies have shown an association of this gene with the risk of developing coronary artery disease [15,16].

In SELL and GUCY1A1 genes, several polymorphisms have been detected, which may affect gene expression.

The aim of our study was to assess the association between the *SELL* rs2205849 and rs2229569 and *GUCY1A1* rs7692387 polymorphisms and the risk of ACS in the form of unstable angina pectoris, and the association between these polymorphisms and selected clinical parameters affecting the risk of developing ischaemic heart disease.

## 2. Materials and Methods

The study included 232 patients with acute coronary syndromes, classified as unstable angina, who required coronary angiography (Table 1). The diagnosis of acute coronary syndromes was achieved by typical clinical presentation and confirmation of significant coronary artery lumen stenosis (>70%) during coronary angiography. Patients with a final diagnosis of MI based on a significant increase in markers of myocardial injury were excluded from the study.

The control group consisted of 144 patients without a history of inflammatory disease or cancer, who underwent coronary angiography for unexplained chest pain (Table 1). In this group of patients, no coronary lumen stenosis was detected via coronary angiography. The study was approved by the ethics committee of the Pomeranian Medical University, Szczecin, Poland (KB-0012/46/17), and written informed consent was obtained from all subjects.

### 2.1. Methods

Genomic DNA was extracted from 1 mL of peripheral blood samples using a Genomic Mini AX Blood 1000 Spin kit (A&A Biotechnology, Gdynia, Poland) following the manufacturer’s protocol. Prior to isolation, blood samples were stored at −80 °C. DNA was subsequently standardised to equal concentrations of 20 ng/µL, based on spectrophotometric absorbance measurements (260/280 nm) (DeNovix DS-11 FX+ Spectrophotometer/Fluorometer, Wilmington, DE, USA).

### 2.2. Genotyping

Genotyping was performed for the following single nucleotide polymorphisms (SNPs): *GUCY1A1* rs7692387, *SELL* rs2205849 and rs2229569 (TaqMan Assay IDs: C__29125113_10, C___2459431_10 and C__25474627_10) using pre-validated allelic discrimination TaqMan real-time PCR assays (Life Technologies, Waltham, MA, USA) and TaqMan GTXpress Master Mix (Life Technologies, Waltham, MA, USA). All reactions were run in duplicate in a final volume of 12 µL (reaction temperature profile: 95 °C for 20 s; followed by 40 cycles of 95 °C for 1 s and 60 °C for 20 s) [17,18].

Fluorescence data were captured using the ViiA7 Real-Time PCR System (Applied Biosystems, Waltham, MA, USA) after a 40-cycle reaction. Genotypes were assigned to individual samples after analysis with TaqMan Genotyper software (Thermo Fisher Scientific, Waltham, MA, USA).

### 2.3. Statistical Analysis

The concordance of genotype distributions with Hardy–Weinberg equilibrium (HWE) was assessed using Fisher’s exact test. The chi-square test was used to compare the distributions of genotypes and alleles between groups. The distribution of quantitative clinical parameters in the study group differed significantly from the normal distribution (Shapiro–Wilk test), thus, they were compared between groups using the non-parametric Mann–Whitney test. A value of *p* < 0.05 was considered statistically significant without correction for multiple testing. The study with 232 patients and 144 controls had sufficient statistical power to detect, with 80% probability, real allelic associations with strength corresponding to odds ratios (OR) of 0.56 or 1.65 for *GUCY1A3* rs7692387, 0.39 or 1.97 for *SELL* rs2205849 and 0.40 or 1.95 for *SELL* rs2229569. Statistical analysis was performed with Statistica 13 and PS Power and Sample Size Calculator (ver. 3.0.43).

## 3. Results

Table 2 presents the distribution of the *SELL* rs2205849 and rs2229569 and *GUCY1A1* rs7692387 polymorphisms in patients with UA and the control group. As shown in Table 2, these differences were not statistically significant. UA patients did not fulfil the HWE for any SNP.

In addition, we performed a comparison of the distribution of the *SELL* rs2205849 and rs2229569 and *GUCY1A1* rs7692387 polymorphisms between patients and control subjects younger and older than 55 years. In the group of patients under 55 years, we found a decreased frequency of the *GUCY1A1* rs7692387AA genotype (AA vs. GA + GG, OR: 0.07; 95% CI: 0.01–0.78) (Table 3).

In the group of patients over 55 years of age, we discovered an increased frequency of the *SELL* rs2205849 CC genotype (CC vs. TC + TT *p* = 0.022) and the *SELL* rs2229569 AA genotype (AA vs. GA + GG *p* = 0.022) (Table 4). 

We also examined the distribution of the *SELL* rs2205849 and rs2229569 and *GUCY1A1* rs7692387 polymorphisms between patients with unstable angina with and without arterial hypertension and diabetes mellitus type 2. As shown in Table 5 and Table 6, these differences were not statistically significant.

The next step of our study was to examine the association between the *SELL* rs2205849, rs2229569 and *GUCY1A1* rs7692387 polymorphisms and selected clinical parameters (BMI, total cholesterol serum levels, high-density cholesterol serum levels, low-density cholesterol serum levels and triacylglycerol serum levels). These associations were not statistically significant (Supplementary Tables S1–S3).

## 4. Discussion

In this study, we examined the association between the *SELL* rs2205849 and rs2229569 and *GUCY1A1* rs7692387 polymorphisms and unstable angina. Analysis of the total study group showed no statistically significant differences between patients and control subjects. However, after dividing the study groups into patients under and over 55 years of age, we found a decreased frequency of the *GUCY1A1* rs7692387AA genotype in the group of patients under 55 years of age. In the group of subjects over 55 years of age, we showed an increased frequency of the *SELL* rs2205849 CC and rs2229569 AA genotypes. The above results suggest that *GUCY1A1* rs7692387AA and *SELL* rs2205849 and rs2229569 polymorphisms are not significant risk factors of acute coronary syndromes under the form of unstable angina in our total population, whereas the effect of these polymorphisms may depend on the age of patients. It seems that the influence of genetic polymorphisms on acute coronary syndromes may depend on various other factors. Previous studies suggest that the effect of a number of atherogenic factors on the development of ischemic heart disease may change with patient age [19,20,21,22,23,24]. This may be related to factors affecting lipid metabolism, the activity of the antioxidant system, platelet function and the activity of vasodilatory and vasoconstrictive mediators. Previous studies have shown a change with age in all of these factors [19,20,21,22,23,24]. Genetic polymorphisms show their impact on coronary heart disease risk together with other atherogenic and pro-inflammatory factors. Thus, it appears that the effect of genetic polymorphisms on the risk of developing coronary artery disease may be age-dependent due to the changing influence of other atherogenic and pro-inflammatory factors with age. Our results also seem to suggest that the effect of GUCY1A1 and SELL gene polymorphisms on the risk of developing unstable angina may depend on the influence of various other factors that change with patient age.

Acute coronary syndromes result from a sudden occlusion of a coronary artery due to rupture or swelling of the atherosclerotic plaque. One of the main causes of this process is inflammation within the vessel and atherosclerotic plaque and the resulting thrombotic changes. Underlying the inflammation is an influx of inflammatory cells, which is exacerbated by the presence of adhesion molecules such as L-selectin [25]. The formation of thrombotic lesions is the responsibility of platelets, whose function, especially their adhesion function, is regulated by many factors, including genetics. Such genes include the *GUCY1A1* gene, which affects platelet adhesion and thrombus formation.

Previous studies have confirmed the role of L-selectin in the inflammatory process, particularly in the recruitment of inflammatory cells [26,27,28,29,30,31]. Population studies have observed an association between adhesion molecule concentrations and age. It was found that subjects over 55 years of age had lower L-selectin serum concentrations than individuals under 55 years of age in both the ischaemic heart disease group and the healthy subject group [32]. Other studies have also found an inverse relationship between age and L-selectin concentration [33,34]. It has been shown that obesity can also be a factor affecting L-selectin levels. Cottam et al. showed that chronic inflammation in subjects with obesity leads to reduced L-selectin levels, affecting neutrophil activation and migration, depressing the immune response and increasing the susceptibility to developing multiple infections [35,36]. Statins have been shown to have a significant effect on lowering serum selectin levels, thereby reducing the risk of cardiovascular events [37].

To date, the association between the *SELL* rs2205849 and rs2229569 polymorphisms and ischaemic heart disease has not been widely studied. Sandoval-Pinto et al. examined *SELL* rs2205849 and rs2229569 polymorphisms in patients with acute coronary symptoms from Mexico. The authors found a decreased frequency of the *SELL* rs2205849 TC and CC genotypes, as well as *SELL* rs2229569 GA and AA in patients with acute coronary symptoms [32]. In our study, we found no statistically significant differences in the *SELL* rs2205849 and rs2229569 polymorphism distribution between the total study and control groups. However, analysing only patients over 55 years of age, we found the increased frequency of the *SELL* rs2205849 CC and rs2229569 AA genotypes among patients with unstable angina. We also found no association of the polymorphisms studied with the risk of hypertension and diabetes, or with the clinical parameters studied.

Another studied SNP was the rs7692387 polymorphism of the *GUCY1A1* gene. The *GUCY1A1* gene encodes the α1 subunit of soluble guanylyl cyclase (sGC), which is a receptor for nitric oxide (NO) that catalyses the formation of the second messenger, cyclic guanosine 3′,5′-monophosphate (cGMP). Through cGMP, nitric oxide exerts vasodilatory, anti-inflammatory and anti-aggregation effects [38]. The appropriate function of nitric oxide ensures proper vascular flow, including in the coronary arteries, and is a factor preventing the development of the atherosclerotic process and conditioning the stability of atherosclerotic plaques, preventing damage. The *GUCY1A1* gene, through its effect on nitric oxide, is a factor that influences vascular function and the risk of developing coronary heart disease and acute coronary syndromes.

Kessler et al. have shown that the *GUCY1A1* rs7692387 polymorphism is located in an intronic site that modulates *GUCY1A1* promoter activity. *GUCY1A1* mRNA levels were significantly lower in dominant homozygotes [15]. Ex vivo studied platelets from GG homozygotes displayed the enhanced inhibition of ADP-induced platelet aggregation by the nitric oxide donor sodium nitroprusside and the phosphodiesterase 5 inhibitor sildenafil compared with individuals who were homozygous for the risk allele. The transcription factor ZEB1 binds preferentially to the A allele, leading to the increased expression of GUCY1A1 and higher levels and activity of soluble guanylyl cyclase after stimulation [15]. The increased expression of soluble guanylyl cyclase is associated with a lower risk of atherosclerosis. These authors have also shown that individuals who are homozygous for the *GUCY1A1* risk allele (GG) displayed increased on-aspirin platelet reactivity compared with individuals with the non-risk allele (AA/AG) [16]. Moreover, homozygous risk allele patients, compared with those with the non-risk allele, were assigned to the high-risk group for ischaemic events. *GUCY1A1* G allele carriers were also at a higher risk of cardiovascular death or stent thrombosis. These authors conclude that individuals who are homozygous for the *GUCY1A1* risk allele are at an increased risk of cardiovascular death or stent thrombosis within 30 days after coronary stenting, likely due to the higher on-aspirin platelet reactivity. The same authors also showed that the G allele is associated with an increased risk of cardiovascular disease and the increased effectiveness of acetylsalicylic acid in its prevention [39]. Emdin et al. showed that the *GUCY1A1* gene rs7692387 polymorphism affects nitric oxide signalling, which is associated with a reduced risk of coronary heart disease, peripheral artery disease and stroke [40]. Li et al. have indicated that *GUCY1A3* rs1842896 polymorphism is an large artery atherosclerotic stroke risk factor in the Southern Han Chinese population [41].

In our study, we did not detect differences in the distribution of SELL rs2205849, rs2229569 and GUCY1A1 rs7692387 polymorphisms between patients with unstable angina and controls. The distribution of these polymorphisms in our Polish population was similar as in other European populations [15,16,39,40]. We found an increased frequency of the GG genotype in patients with unstable angina under 55 years of age. These results suggest that the effect of *GUCY1A3* rs1842896 on the risk of acute coronary syndromes depends on other atherogenic and pro-inflammatory factors that change with the age of the patient. Genetic polymorphisms may be one of many factors influencing the risk of developing coronary artery disease and the occurrence of acute coronary syndromes. The impact of genetic polymorphisms on the development of coronary artery disease may vary in different populations and is dependent on many ethnic and environmental factors. The use of a single polymorphism assay as a predictor of acute coronary syndromes appears to have very limited application in clinical practice. The influence of genetic polymorphisms should only be considered in connection with other factors that increase the risk of developing ischemic heart disease, which may differ between populations as well as change with the age of the patient.

## 5. Conclusions

Our results suggest that the *SELL* rs2205849 and rs2229569 and *GUCY1A1* rs7692387 polymorphisms are not risk factors for unstable angina in the Polish population, and do not significantly affect the risk of developing hypertension and type 2 diabetes. The *GUCY1A1* rs7692387 polymorphism may increase the risk of unstable angina in patients younger than 55 years of age, while the *SELL* rs2205849 and rs2229569 polymorphisms may increase the risk of unstable angina in patients older than 55 years of age in the Polish population.

## Figures and Tables

**Table 1:** Clinical characteristics of patients and control subjects.

Parameters	Control Group *n* = 144	Unstable Angina *n* = 232	*p* *
	Mean ± SD	Mean ± SD	
Age (years)	67.44 ± 10.62	62.07 ± 9.68	<0.00001
BMI (kg/m^2^)	25.96 ± 3.64	28.37 ± 3.95	<0.00001
CH (mg/dL)	197.46 ± 40.98	230.27 ± 56.21	<0.00001
HDL (mg/dL)	53.04 ± 6.77	44.77 ± 8.40	<0.00001
LDL (mg/dL)	118.18 ± 36.84	163.70 ± 50.50	<0.00001
TG (mg/dL)	105.09 ± 45.92	139.77 ± 73.29	<0.00001
	***n* (%)**	***n* (%)**	***p* ˆ**
Sex (male)	54 (37.5%)	172 (74.1%)	<0.0001
Arterial hypertension	57 (39.6%)	145 (62.5%)	<0.0001
Diabetes mellitus	9 (6.3%)	57 (24.6%)	<0.0001

* Mann-Whitney U test; ˆ Fisher’s exact test; BMI—body mass index, CH—total cholesterol in serum, HDL—high-density cholesterol in serum, LDL—low-density cholesterol in serum, TG—triacylglycerols in serum.

**Table 2:** Distribution of *GUCY1A1* rs7692387, *SELL* rs2205849 and rs2229569 genotypes and alleles in UA patients and controls.

	Control Group		Unstable Angina		*p* Value ^^^	Compared Genotypes or Alleles	*p* Value ^#^	OR (95% CI)
	n	%	n	%				
***GUCY1A1 rs7692387*** **genotype**								
GG	91	63.19%	146	62.93%	0.439	AA + GA vs. GG	1.00	1.01 (0.66–1.56)
GA	45	31.25%	79	34.05%		AA vs. GA + GG	0.28	0.53 (0.19–1.49)
AA	8	5.56%	7	3.02%		AA vs. GG	0.28	0.55 (0.19–1.56)
						GA vs. GG	0.73	1.09 (0.70–1.72)
						AA vs. GA	0.26	0.50 (0.17–1.47)
**Allele**								
G	227	78.82%	371	79.96%		A vs. G	0.71	0.93 (0.65–1.34)
A	61	21.18%	93	20.04%				
***SELL*** **rs2205849 genotype**								
TT	119	82.64%	178	76.72%	0.126	CC + TC vs. TT	0.19	1.44 (0.85–2.45)
TC	24	16.67%	45	19.40%		CC vs. TC + TT	0.10	5.77 (0.72–46.04)
CC	1	0.69%	9	3.88%		CC vs. TT	0.10	6.02 (0.75–48.11)
						TC vs. TT	0.49	1.25 (0.73–2.17)
						CC vs. TC	0.16	4.80 (0.57–40.17)
**Allele**								
T	262	90.97%	401	86.42%		C vs. T	0.06	1.58 (0.98–2.57)
C	26	9.03%	63	13.58%				
***SELL*** **rs2229569 genotype**								
GG	118	81.94%	179	77.15%		AA + GA vs. GG	0.30	1. 34 (0.80–2.27)
GA	25	17.36%	44	18.97%	0.152	AA vs. GA + GG	0.10	5.77 (0.72–46.04)
AA	1	0.70%	9	3.88%		AA vs. GG	0.10	5.93 (0.74–47.44)
						GA vs. GG	0.68	1.16 (0.67–2.00)
**Allele**						AA vs. GA	0.15	5.11 (0.61–42.75)
G	261	90.62%	402	86.64%				
A	27	9.38%	62	13.36%		A vs. G	0.11	1.49 (0.92–2.41)

^^^ χ^2^ test; ^#^ Fisher’s Exact Test; HWE: control group *p* = 0.454, unstable angina *p* = 0.417 for *GUCY1A1* rs7692387; HWE: control group *p* = 1.00, unstable angina *p* = 0.020 for *SELL* rs2205849; HWE: control group *p* = 1.00, unstable angina *p* = 0.010 for *SELL* rs2229569.

**Table 3:** Distribution of *GUCY1A1* rs7692387, *SELL* rs2205849 and rs2229569 genotypes and alleles in UA patients and controls in the <55 years group.

	Control Group (*n* = 15)		Unstable Angina (*n* = 55)		*p* Value ^^^	Compared Genotypes or Alleles	*p* Value ^#^	OR (95% CI)
	n	%	n	%				
***GUCY1A1*** **rs7692387** **genotype**								
GG	7	46.67%	34	61.82%	0.025	AA + GA vs. GG	0.38	0.54 (0.17–1.71)
GA	5	33.33%	20	36.36%		AA vs. GA + GG	0.028	0.07 (0.01–0.78)
AA	3	20.00%	1	1.82%		AA vs. GG	0.029	0.07 (0.01–0.76)
						GA vs. GG	0.75	0.82 (0.23–2.94)
						AA vs. GA	0.052	0.08 (0.01–0.98)
**Allele**								
G	19	63.33%	88	80.00%		A vs. G	0.087	0.43 (0.18–1.04)
A	11	36.67%	22	20.00%				
***SELL*** **rs2205849 genotype**								
TT	11	73.33%	41	74.54%	0.872	CC + TC vs. TT	1.00	0.94 (0.26–3.43)
TC	3	20.00%	12	21.82%		CC vs. TC + TT	0.52	0.53 (0.05–6.26)
CC	1	6.67%	2	3.64%		CC vs. TT	0.53	0.54 (0.04–6.48)
						TC vs. TT	1.00	1.07 (0.26–4.48)
						CC vs. TC	1.00	0.50 (0.03–7.54)
**Allele**								
T	25	83.33%	94	85.45%		C vs. T	0.78	0.85 (0.28–2.55)
C	5	16.67%	16	14.55%				
***SELL*** **rs2229569 genotype**								
GG	11	73.33%	41	74.54%		AA + GA vs. GG	1.00	0.94 (0.26–3.43)
GA	3	20.00%	12	21.82%	0.872	AA vs. GA + GG	0.52	0.53 (0.05–6.26)
AA	1	6.67%	2	3.64%		AA vs. GG	0.53	0.54 (0.04–6.48)
						GA vs. GG	1.00	1.07 (0.26–4.48)
**Allele**						AA vs. GA	1.00	0.50 (0.03–7.54)
G	25	83.33%	94	85.45%				
A	5	16.67%	16	14.55%		A vs. G	0.78	0.85 (0.28–2.55)

^^^ χ^2^ test; ^#^ Fisher’s Exact Test.

**Table 4:** Distribution of *GUCY1A1* rs7692387, *SELL* rs2205849 and rs2229569 genotypes and alleles in UA patients and controls in the ≥55 years group.

	Control Group (*n* = 129)		Unstable Angina (*n* = 177)		*p* Value ^^^	Compared Genotypes or Alleles	*p* Value ^#^	OR (95% CI)
	n	%	n	%				
***GUCY1A1 rs7692387*** **genotype**								
GG	84	65.12%	112	63.28%	0.899	AA + GA vs. GG	1.08	0.54 (0.67–1.74)
GA	40	31.01%	59	33.33%		AA vs. GA + GG	1.00	0.87 (0.26–2.92)
AA	5	3.87%	6	3.39%		AA vs. GG	1.00	0.90 (0.27–3.05)
						GA vs. GG	0.71	1.11 (0.68–1.81)
						AA vs. GA	0.76	0.81 (0.23–2.85)
**Allele**								
G	208	80.62%	283	79.94%		A vs. G	0.92	1.04 (0.70–1.56)
A	50	19.38%	71	20.06%				
***SELL*** **rs2205849 genotype**								
TT	108	83.72%	137	77.40%	0.057	CC + TC vs. TT	0.19	1.50 (0.84–2.70)
TC	21	16.28%	33	18.64%		CC vs. TC + TT	0.022	-
CC	0	0.00%	7	3.96%		CC vs. TT	0.021	-
						TC vs. TT	0.55	1.24 (0.68–2.26)
						CC vs. TC	0.08	-
**Allele**								
T	237	91.86%	307	86.72%		C vs. T	0.051	1.73 (1.01–2.97)
C	21	8.14%	47	13.28%				
***SELL*** **rs2229569 genotype**								
GG	107	82.95%	138	77.96%		AA + GA vs. GG	0.31	1.38 (0.77–2.46)
GA	22	17.05%	32	18.08%	0.068	AA vs. GA + GG	0.022	-
AA	0	0.00%	7	3.96%		AA vs. GG	0.021	-
						GA vs. GG	0.76	1.13 (0.62–2.05)
**Allele**						AA vs. GA	0.042	-
G	236	91.47%	308	87.01%				
A	22	8.53%	46	12.99%		A vs. G	0.09	1.60 (0.94–2.74)

^^^ χ^2^ test; ^#^ Fisher’s Exact Test.

**Table 5:** Distributions of the *SELL* rs2229569, rs2205849 and *GUCY1A1* rs7692387 genotypes and alleles in UA patients with and without diabetes mellitus (DM).

	Without		Diabetes		*p* Value ^^^		*p* Value *	OR (95% CI)
	Diabetes		Mellitus					
	Mellitus							
	*n*	%	*n*	%				
***SELL*** **rs2229569**								
**genotype**								
GG	134	76.57%	45	78.95%	0.63	AA + GA vs. GG	0.86	0.87 (0.42–1.80)
GA	33	18.86%	11	19.30%		AA vs. GA + GG	0.46	0.37 (0.05–3.05)
AA	8	4.57%	1	1.75%		AA vs. GG	0.46	0.37 (0.05–3.06)
						GA vs. GG	1.00	0.99 (0.46–2.13)
						AA vs. GA	0.67	0.38 (0.04–3.34)
**Allele**								
G	301	86.00%	101	88.60%		A vs. G	0.53	0.79 (0.41–1.52)
A	49	14.00%	13	11.40%				
***SELL***								
**rs2205849**								
**genotype**								
TT	133	76.00%	45	78.95%	0.63	CC + TC vs. TT	0.72	0.84 (0.41–1.74)
TC	34	19.43%	11	19.30%		CC vs. TC + TT	0.46	0.37 (0.05–3.05)
CC	8	4.57%	1	1.75%		CC vs. TT	0.46	0.37 (0.05–3.04)
						TC vs. TT	1.00	0.96 (0.45–2.04)
						CC vs. CT	0.67	0.39 (0.04–3.44)
**Allele**								
T	300	85.71%	101	88.60%		C vs. T	0.53	0.77 (0.40–1.48)
C	50	14.29%	13	11.40%				
***GUCY1A1***								
**rs7692387**								
**genotype**								
GG	108	61.71%	38	66.67%	0.70	AA + GA vs. GG	0.53	0.81 (0.43–1.51)
GA	61	34.86%	18	31.58%		AA vs. GA + GG	1.00	0.50 (0.06–4.27)
AA	6	3.43%	1	1.75%		AA vs. GG	0.68	0.47 (0.06–4.06)
						GA vs. GG	0.63	0.84 (0.44–1.60)
						AA vs. GA	1.00	0.57 (0.06–5.00)
**Allele**								
G	277	79.14%	94	82.46%		A vs. G	0.50	0.81 (0.47–1.40)
A	73	20.86%	20	17.54%				

^^^ χ2 test; * Fisher’s exact test.

**Table 6:** Distributions of the *SELL* rs2229569, rs2205849 and *GUCY1A1* rs7692387 genotypes and alleles in UA patients with and without arterial hypertension (HA).

	Without		Arterial		*p* Value ^^^		*p* Value *	OR (95% CI)
	Arterial		Hypertension					
	Hypertension							
	*n*	%	*n*	%				
***SELL*** **rs2229569**								
**genotype**								
GG	67	77.01%	112	77.24%	0.14	AA + GA vs. GG	1.00	0.99 (0.52–1.86)
GA	14	16.09%	30	20.69%		AA vs. GA + GG	0.08	0.29 (0.07–1.17)
AA	6	6.90%	3	2.07%		AA vs. GG	0.09	0.30 (0.07–1.24)
						GA vs. GG	0.60	1.28 (0.64–2.59)
						AA vs. GA	0.07	0.23 (0.05–1.07)
**Allele**								
G	148	85.06%	254	87.59%		A vs. G	0.48	0.81 (0.47–1.39)
A	26	14.94%	36	12.41%				
***SELL***								
**rs2205849**								
**genotype**								
TT	66	75.86%	112	77.24%	0.16	CC + TC vs. TT	0.87	0.93 (0.50–1.73)
TC	15	17.24%	30	20.69%		CC vs. TC + TT	0.08	0.29 (0.07–1.17)
CC	6	6.90%	3	2.07%		CC vs. TT	0.09	0.30 (0.07–1.22)
						TC vs. TT	0.73	1.18 (0.60–2.35)
						CC vs. CT	0.13	0.25 (0.06–1.14)
**Allele**								
T	147	84.48%	254	87.59%		C vs. T	0.40	0.77 (0.45–1.32)
C	27	15.52%	36	12.41%				
***GUCY1A1***								
**rs7692387**								
**genotype**								
GG	56	64.37%	90	62.07%	0.46	AA + GA vs. GG	0.78	1.10 (0.64–1.92)
GA	27	31.03%	52	35.86%		AA vs. GA + GG	0.43	0.44 (0.10–2.01)
AA	4	4.60%	3	2.07%		AA vs. GG	0.43	0.47 (0.10–2.16)
						GA vs. GG	0.57	1.20 (0.68–2.12)
						AA vs. GA	0.25	0.39 (0.08–1.87)
**Allele**								
G	139	79.89%	232	80.00%		A vs. G	1.00	0.99 (0.62–1.59)
A	35	20.11%	58	20.00%				

^^^ χ2 test; * Fisher’s exact test.

## Data Availability

Not applicable.

## Supplementary Materials

The following supporting information can be downloaded at: www.mdpi.com/xxx/s1.Table S1. Associations between the clinical parameters of patients with unstable angina and the *SELL* rs2229569 genotypes; Table S2. Associations between the clinical parameters of patients with unstable angina and the *SELL* rs2205849 genotypes, Table S3. Associations between the clinical parameters of patients with unstable angina and the *GUCY1A1* rs7692387genotypes.
