# Supplementary material for: SELL and GUCY1A1 Gene Polymorphisms in Patients with Unstable Angina
Source: Biomedicines. 2022 Oct 6;10(10):2494. doi: 10.3390/biomedicines10102494 (PMC9598954; doi:10.3390/biomedicines10102494)
Supplement: Supplementary file 1 [file biomedicines-10-02494-s001.zip › biomedicines-1906387-supplementary.pdf]

biomedicines

MDPI

SELL and *GUCY1A1* gene polymorphisms in patients with unstable angina

Damian Malinowski, Magda Zawadzka, Krzysztof Safranow, Marek Droździk and Andrzej Pawlik

Table S1. Associations between the clinical parameters of patients with unstable angina and the *SELL* rs2229569 genotypes.

Parameters	*SELL* rs2229569 genotype												
	GG		GA		AA		GG+GA		GA+AA		GG vs. GA+AA	GG+GA vs. AA	GG vs. AA
	n	Mean ± SD	*n*	Mean ± SD	*n*	Mean ± SD	*n*	Mean ± SD	*n*	Mean ± SD	p [&]		
Age [years]	179	62.39 ± 9.49	44	60.93 ± 10.72	9	61.22 ± 8.53	223	62.1 ± 9.74	53	60.98 ± 10.31	0.433	0.753	0.676
BMI [kg/m^2]	179	28.33 ± 3.90	44	28.98 ± 4.07	9	26.22 ± 4.09	223	28.46 ± 3.93	53	28.51 ± 4.17	0.959	0.136	0.152
CH [mg/dL]	171	230.48 ± 57.69	43	230.00 ± 51.04	9	227.67 ± 57.21	214	230.38 ± 56.30	52	229.60 ± 51.57	0.861	0.868	0.852
HDL [mg/dL]	139	44.89 ± 8.20	39	44.21 ± 9.34	8	45.5 ± 8.02	178	44.74 ± 8.44	47	44.43 ± 9.06	0.536	0.742	0.798
LDL [mg/dL]	139	164.22 ± 51.22	39	162.31 ± 47.75	8	161.5 ± 57.16	178	163.80 ± 50.36	47	162.17 ± 48.79	0.902	0.947	0.912
TG [mg/dL]	170	134.30 ± 60.69	43	158.86 ± 110.30	8	151.78 ± 62.79	213	139.26 ± 73.79	52	157.64 ± 103.17	0.240	0.433	0.371

[&]—Mann–Whitney U test; BMI—body mass index; CH—total cholesterol in serum; HDL—high density cholesterol in serum; LDL—low density cholesterol in serum; TG—triacylglycerols in serum.

Table S2. Associations between the clinical parameters of patients with unstable angina and the *SELL* rs2205849 genotypes.

Parameters	*SELL* rs2205849 Genotype												
	TT		TC		CC		TT+TC		TC+CC		TT vs. TC+CC	TT+TC vs. CC	TT vs. CC
	n	Mean ± SD	*n*	Mean ± SD	*n*	Mean ± SD	*n*	Mean ± SD	*n*	Mean ± SD	p [&]		
Age [years]	178	62.40 ± 9.51	45	60.89 ± 10.61	9	61.22 ± 8.53	223	62.1 ± 9.74	54	60.94 ± 10.22	0.335	0.754	0.672
BMI [kg/m^2]	178	28.31 ± 3.90	45	29.04 ± 4.05	9	26.22 ± 4.09	223	28.46 ± 3.93	54	28.57 ± 4.16	0.823	0.137	0.157

CH [mg/dL]	170	230.73 ± 57.76	44	229.00 ± 50.88	9	227.67 ± 57.21	214	230.38 ± 56.30	53	228.77 ± 51.42	0.974	0.868	0.848
HDL [mg/dL]	138	44.94 ± 8.21	40	44.05 ± 9.27	8	45.5 ± 8.02	178	44.74 ± 8.44	48	44.29 ± 9.01	0.444	0.742	0.816
LDL [mg/dL]	138	164.54 ± 51.27	40	161.25 ± 47.60	8	161.5 ± 57.16	178	163.80 ± 50.36	48	161.29 ± 48.65	0.770	0.947	0.904
TG [mg/dL]	169	134.60 ± 60.74	44	157.14 ± 109.60	9	151.78 ± 62.79	213	139.26 ± 73.79	53	156.23 ± 102.69	0.314	0.433	0.381

&—Mann–Whitney U test; BMI—body mass index; CH—total cholesterol in serum; HDL—high density cholesterol in serum; LDL—low density cholesterol in serum; TG—triacylglycerols in serum.

Table S3. Associations between the clinical parameters of patients with unstable angina and the *GUCY1A1 rs7692387*genotypes.

**Parameters**	***GUCY1A1* rs7692387 Genotype**												
	**GG**		**GA**		**AA**		**GG+GA**		**GA+AA**		**GG vs. GA+AA**	**GG+GA vs. AA**	**GG vs. AA**
	n	**Mean ± SD**	***n***	**Mean ± SD**	***n***	**Mean ± SD**	***n***	**Mean ± SD**	***n***	**Mean ± SD**	**p &**		
Age [years]	146	62.10 ± 9.66	79	61.84 ± 9.93	7	63.86 ± 8.05	225	62.01 ± 9.73	86	62.00 ± 9.76	0.998	0.631	0.625
BMI [kg/m^2]	146	28.56 ± 3.99	79	28.09 ± 3.93	7	27.71 ± 3.86	225	28.39 ± 3.96	86	28.06 ± 3.90	0.351	0.934	0.962
CH [mg/dL]	139	228.37 ± 58.38	77	232.39 ± 52.96	7	244.86 ± 51.06	216	229.80 ± 56.14	84	233.43 ± 52.61	0.288	0.293	0.247
HDL [mg/dL]	113	44.36 ± 8.06	69	45.62 ± 8.93	4	41.50 ± 9.15	182	44.84 ± 8.40	73	45.40 ± 8.93	0.529	0.338	0.384
LDL [mg/dL]	113	163.79 ± 52.21	69	162.81 ± 48.92	4	176.75 ± 31.38	182	163.42 ± 50.85	73	163.58 ± 48.08	0.793	0.372	0.384
TG [mg/dL]	139	140.17 ± 75.27	76	138.30 ± 71.88	7	147.71 ± 53.43	215	139.51 ± 73.93	83	139.10 ± 70.29	0.854	0.475	0.461

&—Mann–Whitney U test; BMI—body mass index; CH—total cholesterol in serum; HDL—high density cholesterol in serum; LDL—low density cholesterol in serum; TG—triacylglycerols in serum.
